# The Ionospheric Connection Explorer - Prime Mission Review

**DOI:** 10.1007/s11214-023-00975-x

**Published:** 2023-07-17

**Authors:** Thomas J. Immel, Scott L. England, Brian J. Harding, Yen-Jung Wu, Astrid Maute, Chihoko Cullens, Christoph R. Englert, Stephen B. Mende, Roderick A. Heelis, Harald U. Frey, Eric J. Korpela, Andrew W. Stephan, Sabine Frey, Michael H. Stevens, Jonathan J. Makela, Farzad Kamalabadi, Colin C. Triplett, Jeffrey M. Forbes, Emma McGinness, L. Claire Gasque, John M. Harlander, Jean-C. Gérard, Benoit Hubert, Joseph D. Huba, Robert R. Meier, Bryce Roberts

**Affiliations:** 1 Space Sciences Laboratory, University of California, Berkeley, 7 Gauss Way, Berkeley, 94720-7450 CA USA; 2 Aerospace and Ocean Engineering, Virginia Polytechnic Institute and State University, Blacksburg, VA 24061 USA; 3 CIRES, University of Colorado, Boulder, CO 80309 USA; 4 Laboratory for Atmospheric and Space Physics, Univ. of Colorado, Boulder, TX 80309 USA; 5 U.S. Naval Research Laboratory, 4555 Overlook Ave S.W., Washington, DC 20375 USA; 6 William B. Hanson Center for Space Sciences, University of Texas, Dallas, Richardson, TX 75080 USA; 7 Department of Electrical and Computer Engineering, University of Illinois Urbana-Champaign, Urbana, IL 61801 USA; 8 Department of Aerospace Engineering Sciences, University of Colorado, Boulder, CO 80303 USA; 9 Space Systems Research Corporation, Alexandria, VA USA; 10 University of Liège, Liège, Belgium; 11 Syntek Technologies, Arlington, VA USA; 12 George Mason University, Fairfax, VA USA

**Keywords:** Ionosphere, Thermosphere, Ion-neutral coupling, Mesosphere

## Abstract

The two-year prime mission of the NASA Ionospheric Connection Explorer (ICON) is complete. The baseline operational and scientific objectives have been met and exceeded, as detailed in this report. In October of 2019, ICON was launched into an orbit that provides its instruments the capability to deliver near-continuous measurements of the densest plasma in Earth’s space environment. Through collection of a key set of in-situ and remote sensing measurements that are, by virtue of a detailed mission design, uniquely synergistic, ICON enables completely new investigations of the mechanisms that control the behavior of the ionosphere-thermosphere system under both geomagnetically quiet and active conditions. In a two-year period that included a deep solar minimum, ICON has elucidated a number of remarkable effects in the ionosphere attributable to energetic inputs from the lower and middle atmosphere, and shown how these are transmitted from the edge of space to the peak of plasma density above. The observatory operated in a period of low activity for 2 years and then for a year with increasing solar activity, observing the changing balance of the impacts of lower and upper atmospheric drivers on the ionosphere.

## Introduction

The Ionospheric Connection Explorer (ICON) is a NASA Explorer, selected for development on April 12, 2013. ICON was launched more than 6 years later at 21:39 Eastern Daylight Time on October 10, 2019 from Cape Canaveral Air Force Station on a Pegasus XL launch vehicle dropped by the Northrop Grumman Stargazer L-1011 carrier aircraft. After on-orbit checkout ICON entered a 2-year period of prime mission science operations on December 15, 2019. The observatory was operated successfully for almost three years, with a final contact made by the ground station in Santiago, Chile on November 25 2022, ending at 15:00 UT, followed by an unexplained failure to communicate using the same ground station two orbits later. No attempts to command the spacecraft made at any time thereafter appeared to be successful. Thus, even with an abrupt and unexpected end to a flight mission that was to that point exhibiting only good performance, ICON collected more than 1000 days of observations and exceeded its performance requirements. This report is the first of a series that focuses on each instrument and each scientific retrieval to fully characterize the scientific capability that the publicly-available data reflect. Key parameters of the ICON orbit and observatory are reported in Table 1.

**Table 1:** Mission Facts

Parameter	Value
Inclination	27 deg.
Precession period	48 days^a^
Insertion Orbit	611 km × 584 km
Current Orbit	603 km × 576 km^b^
Launch Mass	288 kg
	*Payload* 130 kg
	*Spacecraft* 158 kg
Observatory Power Use	289 W^c^

^a^Full Local Time Coverage.

^b^Nov. 2022.

^c^Orbit Average, *β* = 0.

### Scientific Objectives and Requirements

The ICON mission (Immel et al. 2018) has three main scientific objectives; 1. Finding the cause of day-to-day variability in the ionosphere, 2. Determining the key drivers of seasonal changes in the ionosphere, and 3. Understanding the competing influences of geomagnetic storms as they modify the ionosphere. Immel et al. (2018) describe these objectives in significant detail. The scientific requirements of the mission were developed to address these objectives. These define the local time and altitude range, precision and spatio-temporal resolution with which specific key parameters of the ionosphere and thermosphere are to be retrieved. These requirements are described completely in the initial mission design report (Immel et al. 2018), where the scientific requirements at both the Program Level and Project Level (where performance margin vs Program requirements is held) are discussed in detail. Below we report in tabular form (Table 2) the qualities of the data products that are required to address the objectives listed above.

**Table 2:** Required per sample performance for each ICON data product. Products 2.1, 2.2 and 2.3 were retrieved using 30 s (60 s) sampling in day (night); retrievals for products 2.4-2.6 used 12 s sampling. Product 2.7 was determined every 4 s with required precision

ICON Data Products					
Level 2 product number	Key Parameter	Local Time	Altitude Range (km)	Vertical Resolution	Required Precision
**2.1, 2.2**	Horizontal Winds; Line of sight and cardinal vectors	Day	105-150	5 km	10 m/s
		Night	200-300	30 km	8.7 m/s
		Both	90-105	5 km	8.7 m/s
		Day	170-200	30 km	10 m/s
**2.3**	Neutral Temperatures	Day, Night	90-105	5 km	12.4 K
**2.4**	O/N_2_ Ratio	Day	N/A^a,b^		8.7%
**2.5**	O^+^ Profile	Night	200-400	10 km	10% @ F-peak
**2.6**	O^+^ Profile	Day	200-400	10 km	10% @ F-peak
**2.7**	Plasma Velocity	6-24 hr	in-situ		7.5 m/s

^a^Column integrated quantity.

^b^Imager provides vertical resolution on Earth’s limb of 8 km altitude (Mende et al. 2017).

The science team also defined the required orbit parameters for making the observations and, for the required measurements, developed a set of seven key data products using five separate retrieval algorithms, all of which have been validated and collectively brought to bear on each of the objectives. The ICON team released the first scientific data collected during the mission’s first six months to the public on June 22, 2020, and continues regular public release of all data products.

Using these products to address its top objective, ICON showed that variations in the ionospheric wind dynamo (Richmond et al. 1976; Richmond 1991; Maute 2021) are a key source of day-to-day variability in the ionosphere (Science Objective 1). This is based on correlations determined between instantaneous observations of vertical plasma drift at the equator and values predicted using simultaneous E-region wind measurements on the same magnetic field lines. Because the global structure in these winds is largely introduced by tides originating below 30 km altitude, this finding is a remarkable demonstration of the strong influence that conditions in the lower atmosphere exert on conditions in the equatorial ionosphere.

Objective 2 has been fulfilled using retrievals of atmospheric tides based upon both the simultaneous wind and temperature measurements that ICON provides continuously during day and night at the boundary of space. These provide the basis for a complete evaluation of the importance of day-to-day tidal variations (Forbes et al. 2021), production of a Hough Mode Extension (HME) product that characterizes the atmospheric tides (Cullens et al. 2020), and a mission-length general circulation simulation (TIEGCM (Roble and Ridley 1994)) that uses the HME product to inform its lower boundary. Analysis of the other instrument products finds how each of the other key parameters in the ITM system respond, or mediate the response, to atmospheric tides.

The observation of geomagnetic storm effects during deep solar minimum was one of the most remarkable scientific activities contributing to science objective 3, showing at once the high sensitivity of the IT system to even minor storm-time inputs, and the broad range of effects observed for seemingly similar inputs of small storms and substorms. Analysis of data from the prime mission shows also that storm-driven changes are often of the same magnitude as day-to-day variability driven by terrestrial weather. The corresponding papers are in work and highlights are included in this review article.

### Science Payload

The observatory carries a science payload of four instruments to provide the above noted data products.

1. The Michelson Interferometer for Global High-resolution Thermospheric Imaging (MIGHTI) (Englert et al. 2017) provides the limb measurements in visible light used for retrieval of data products 2.1-2.3. It measures Doppler shift of 630.0 and 557.7-nm red and green atmospheric airglow from which neutral winds between 90-300 km are retrieved. The lower portion of the red channel images the 762-nm atmospheric O_2_ band to retrieve temperatures at altitudes spanning the boundary of space. On orbit, this instrument worked well with no anomalies. The latest wind products (version 5) released after November 2022 include algorithm updates informed by the actual on-orbit performance of the instrument and calibrations, which has the greatest importance for red-line measurements above 160 km and at night. Specifically, this includes the zero-wind calibrations. Gaps in the product of up to several days are due to remaining issues of solar and lunar contamination and continue to be addressed.
2. The Far Ultraviolet imaging spectrograph (FUV) (Mende et al. 2017) provides measurements of FUV emissions on both Earth’s limb and disk to support retrieval of data products 2.4 and 2.5. Its two channels measure atomic oxygen (O) emissions at 135.6 nm and molecular nitrogen (N_2_) emissions at 157 nm. FUV measurements taken under sunlit conditions provide the atmospheric O/N_2_ ratio, while measurements of the nighttime 135.6-nm recombination emission provide vertical profiles of O^+^ ion density. On orbit, higher-than-expected levels of stray light in the long wave channel were addressed by using data collected below the horizon to produce the required thermospheric column O/N_2_ product (as TIMED GUVI (Christensen et al. 2003)). The final limb product that implements an instrument scattering function determined from stellar observations is currently in verification and will provide a second O/N_2_ product.
3. The Extreme Ultraviolet (EUV) spectrograph (Sirk et al. 2017) provides limb measurements required for product 2.6. It measures the 61.7-nm and 83.4-nm emissions associated with ionized oxygen (O^+^), from which the daytime O^+^ density can be inferred. On orbit it performed well and allowed for the retrieval as planned. After 2 years the detector required an increase in high voltage, and the performance of the instrument in this new configuration was characterized by successive observations of the full moon. The instrument was set to a higher voltage and then set to make observations at a lower duty cycle (∼20%) to validate the performance of the ionospheric retrieval with the new setting. The planned ramp up to full duty observations was not implemented before the mission end in November 2022.
4. The Ion Velocity Meter (IVM) (Heelis et al. 2017) provides measurements to determine product 2.7 - ion velocity and other properties of the plasma including ion temperature, the total ion number density and the relative abundance of O^+^ and H^+^ ions. Two units allow for full science data collection in normal and reversed flight orientations (described in Sect. 1.3) On orbit, both units performed similarly and well. In the first ∼18 months of the mission the low plasma densities encountered at solar minimum combined with solar illumination of the aperture negatively affected drift velocity determinations at morning local times. The instrument otherwise performed well when measuring velocities in the afternoon and at night. By the end of the mission the observed increase in solar EUV fluxes produced higher plasma densities that mitigated the issues with solar illumination of the aperture, allowing for plasma velocity determinations at all local times.

Each of the three remote-sensing limb imagers requires the implementation of scientific algorithms to retrieve the physical quantities of the atmosphere and ionosphere from the radiance information that it provides. The retrieval algorithm theory and predicted performance are described in separate review articles for the wind and temperature retrievals (Harding et al. 2017; Stevens et al. 2018), the daytime thermospheric composition retrieval (Stephan et al. 2018), the nighttime ionospheric density retrieval (Kamalabadi et al. 2018), and the daytime ionospheric density retrieval (Stephan et al. 2017). The on-orbit performance of those retrievals and the IVM instrument are reported in this journal (Englert et al. 2023; Heelis et al. 2022; Stevens et al. 2022; Wautelet et al. 2022).

### Observatory Performance

The observatory carried remarkable capability in terms of power generation (>700 W solar array production capability), pointing knowledge and control (<0.01^∘^), and authority to rapidly adjust pitch, yaw, and roll. Rapid rotations of the satellite provided for quickly performed calibration maneuvers and for pointing exercises that expanded the scientific capability of the mission. For example, the operation we describe as the conjugate maneuver provides wind measurements at both magnetic footpoints of the observatory as it crosses the magnetic equator. This is described in more detail in a following section. Another capability that was enabled by the ability to rapidly rotate the observatory is the “zero-wind” maneuver. This aimed the field of view of either of the MIGHTI channels to the limb in the orbit track, first ahead of the observatory, and then behind. This provided two independent measurements of the same region and, under the assumption that the wind velocity does not change in ∼10 minutes, allows the calculation of the position of the interferometer fringes that represents a Doppler shift of light from a source stationary relative to the instrument (“zero wind”). This provides a means to produce a wind product with accuracy on the order of the instrument precision. Though high accuracy winds are not a mission requirement, the capability simplifies comparisons to other wind measurements and thermospheric wind models.

During the two-year science mission, the observatory experienced four anomalies related to star-tracker outages, and the periods of these anomalies are reported in Table 3. These anomalies occurred during periods of high orbital beta angle, where the temperature of the trackers were relatively high and the associated noise levels interfered with star tracking in daytime. In worst cases the star-tracker controlling software would not recover and the onboard orbit propagator would report a lack of updates. When that condition persisted for a set number of minutes, the spacecraft would respond to by safing the science payload and transitioning to a sun-tracking mode. These anomalies occurred near peaks in $\beta $ around June solstice when it approached 50^∘^. The issue was identified and mitigated in 2021 with updated tracker firmware and a software monitor installed on the spacecraft to evaluate the state of the tracker unit and power-cycle it prior to a watchdog-initiated reset of the flight computer. One nuance is that the second of these events, in order to avoid another failure of the star tracker under high orbit beta conditions, the observatory was recovered to a local-vertical local-horizontal (LVLH) orientation with a 180-degree yaw from nominal flight, a configuration we call reverse LVLH (rLVLH). This configuration places the FOVs of the star trackers (and the remote sensing instruments) southward where they remain cooler and not susceptible to solar radiance inputs around June solstice. IVM-B is then tasked to provide the in-situ plasma data. This alternate mode of flight is a valid science configuration for the mission that can provide all baseline data products. After all the star tracker patches, this was no longer necessary at high beta, but remained a useful option for science until the end of the mission. No star-tracker upsets were observed after September 2021.

**Table 3:** Observatory Safe Modes

Start Time	End Time
Feb 19 2020 16:00 UT	Feb 25 2020 00:00 UT
April 29 2021 4:09 UT	May 5 2021 00:25 UT
June 13 2021 12:25 UT	June 16 2021 03:48 UT^a,b^
Sept 2 2021 20:19 UT	Sept 8 2021 18:30 UT

^a^Recovery to reverse LVLH.

^b^Return to LVLH June 27, 2021 01:50 UT.

Apart from these events the spacecraft power systems, communications, attitude control and thermal systems performed nominally until the end of mission. ICON carried no propulsion elements or other consumables, and at the end of mission the observatory power system was healthy, supporting baseline science operations at all local times, and power positive in science mode at all beta angles (±50). ICON’s orbit will provide for natural re-entry prior to the 25-year required limit.

To meet its Baseline Level 1 requirements, ICON needed to provide scientific observations from all four of its instruments for four seasons. Seasons are defined as periods of 91 days, from which data in 60 days would provide the sampling of that season. All instruments were fully checked out and in science mode by December 15th, 2019. The brief outages in science data collection associated with the anomalies related to the star-tracker were short-lived relative to this 60-of-91-day Level 1 requirement. Thus, by December 16th, 2020 ICON had obtained all the data to meet its Level 1 requirements. The data collected during the remainder of the Prime Mission has significantly enhanced the scientific return of the mission, adding to the parameter space of conditions observed. This includes observing more periods of enhanced geomagnetic activity during which ICON can act as a pathfinder for new science investigations to be performed during the upcoming NASA Geospace Dynamics Constellation and DYNAMIC missions (see Sect. 3.3).

The observational science state, either in LVLH or rLVLH configuration, is interrupted by specific lunar, stellar, and nadir pointing states to support calibration of the science instruments, or the aforementioned calibration maneuvers for MIGHTI. The number of calibration modes set by ICON during the entire science mission (December 2019 to November 2022) is shown in Table 4. Conjugate maneuvers are also shown. Note that there are instrument-level calibration steps/modes that do not require maneuvers and are therefore not included in this table.

**Table 4:** Observatory Maneuvers^a^

Calibration Maneuver	Number Performed
Conjugate	359
Zero-wind calibration	77
Nadir calibrations	45
FUV star calibrations	41
EUV lunar calibrations	48
Reverse LVLH	3

^a^Calibrations are all approximately 10-15 minutes in duration.

## New Observations

**Fig. 1:**
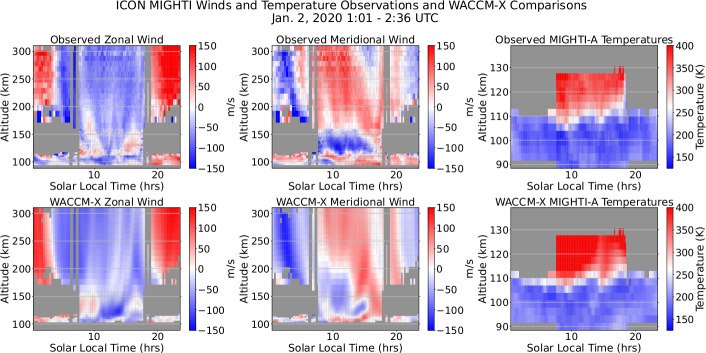
MIGHTI wind and temperature measurements for a single orbit on January 2, 2022 are shown in the top row. The bottom row shows the comparable winds and temperatures from WACCM-X run. With some clear exceptions, a great deal of the large-scale structure observed during this orbit is represented by WACCM-X

A number of new capabilities have been realized by ICON. Some of the most remarkable findings of the ICON mission come from ICON/MIGHTI that provides, for the first time, wind profiles from 90-300 km in the daytime thermosphere, which are continuous in altitude along the orbit track. Through the use of an Abel retrieval algorithm (Harding et al. 2017) applied to the interferometric phases and amplitudes detected in the MIGHTI instrument and reported in the calibrated Level 1 MIGHTI product (Harlander et al. 2017), ICON has revealed remarkable variability and intense wind shears in the thermosphere. An example of a single orbit of observations (Fig. 1) shows the capability of the MIGHTI instrument for retrieving thermospheric winds in both day and night (left and center top). This cardinal wind product (2.2) is retrieved in combining the two independent channels of MIGHTI that make independent Line-of-Sight (LoS) measurements that are combined with a time offset for when they are perpendicular at the same tangent point on Earth’s limb. These winds are accompanied by temperature measurements in the lower altitude ranges with the two channels now acting independently, showing MIGHTI-A temperatures in the right top of Fig. 1. These measurements together effectively capture the propagation of waves into the lower thermosphere from the atmosphere below as they modify the local environment. For comparison, the same wind and temperature fields from a simulation using the Whole Atmosphere Community Climate Model with thermosphere and ionosphere extension (Liu et al. 2018; Hsu and Pedatella 2021) run under solar quiet conditions are shown at the same locations and times as the MIGHTI observations in the bottom row of Fig. 1. There is signficant agreement between the modeled winds and observations, with generally best comparisons in the zonal direction for this orbit. The temperatures in the model show high fidelity to the measured temperatures. A broader review of the model vs. data show some more significant variation in the fidelity of the model to the observations from one day to the next.

MIGHTI measurements are also supporting a reexamination of the TIMED TIDI winds (Dhadly et al. 2021; Wu and Ridley 2023) which continue to be collected in the 20th year of the TIMED mission. The wind products have been verified in comparisons to simultaneously-obtained ground-based interferometric Doppler observations of thermospheric winds determined from the same nighttime oxygen red-line emissions at 630.0 nm (Makela et al. 2021) observed by MIGHTI. The ICON wind products determined from the green line oxygen emission at 557.7 nm near 100 km have been shown to compare well to the same winds determined from ground-based meteor radars in both day and night (Harding et al. 2021). MIGHTI wind measurements also have been used to validate mesospheric wind profiles measured in solar occultation experiments by the ACE-FTS on SCISAT (Boone et al. 2021). The temperature and wind products are both now at version 5 and available through August 2023 with efforts now being made to finalize through November and end of mission.

MIGHTI temperature retrievals (Stevens et al. 2018) have been shown to be in agreement with ground-based retrievals of mesospheric temperatures using lidar techniques (Yuan et al. 2021; Wing et al. 2021). An extensive comparison to temperature retrievals from the SABER instrument flying on the extended TIMED mission (Christensen et al. 2003) using over 1400 instances of common volume measurements between 90 and 110 km for both MIGHTI-A and -B is discussed in this journal by Stevens et al. (Stevens et al. 2022). In that report it is shown that the MIGHTI and SABER temperature retrievals consistently compare well, and the apparent high bias of SABER temperatures relative to MIGHTI is mostly consistent with the combined uncertainties reported in the SABER and MIGHTI temperature products.

The combined tidal product, the Hough Mode Extension (Forbes et al. 2017; Cullens et al. 2020), which is informed by both MIGHTI winds and temperatures, has been used to explain the formation of metal ion layers in the lower thermosphere at nighttime (Chu et al. 2021), with another study by Yamazaki *et al.* using the wind measurements directly to investigate the physics of the development of sporadic-E layers (Yamazaki et al. 2022). The winds and temperatures from ICON are widely used and will continue to have a major scientific impact now and into the future.

FUV observations provide both daytime retrievals of the thermospheric oxygen to nitrogen ratio (Stephan et al. 2018; Meier 2021), as well as nighttime O^+^ density profiles (Kamalabadi et al. 2018). The means by which the nighttime data are collected implements both a steerable field-of-view (FOV) and a time-delay imaging (TDI) technique such that large depletions in ionospheric density are resolved in the most advantageous manner possible (Wilkins et al. 2017). The turret orients the FOV along the magnetic field and the TDI processing provides a velocity correction while integrating the signal collected while imaging the remote scene, effectively compensating for the motion of the observatory during every 12-s integration. This is done only for the O 135.6-nm channel, where emissions are produced at night in the process of recombination of O^+^. An update to the nighttime product is in work, expected to be released later in 2023, where a notification flag will be used to identify retrievals where requirements of spherical symmetry in the radiance scene are violated, producing uncertainties larger than can be characterized by the retrieval algorithm itself.

**Fig. 2:**
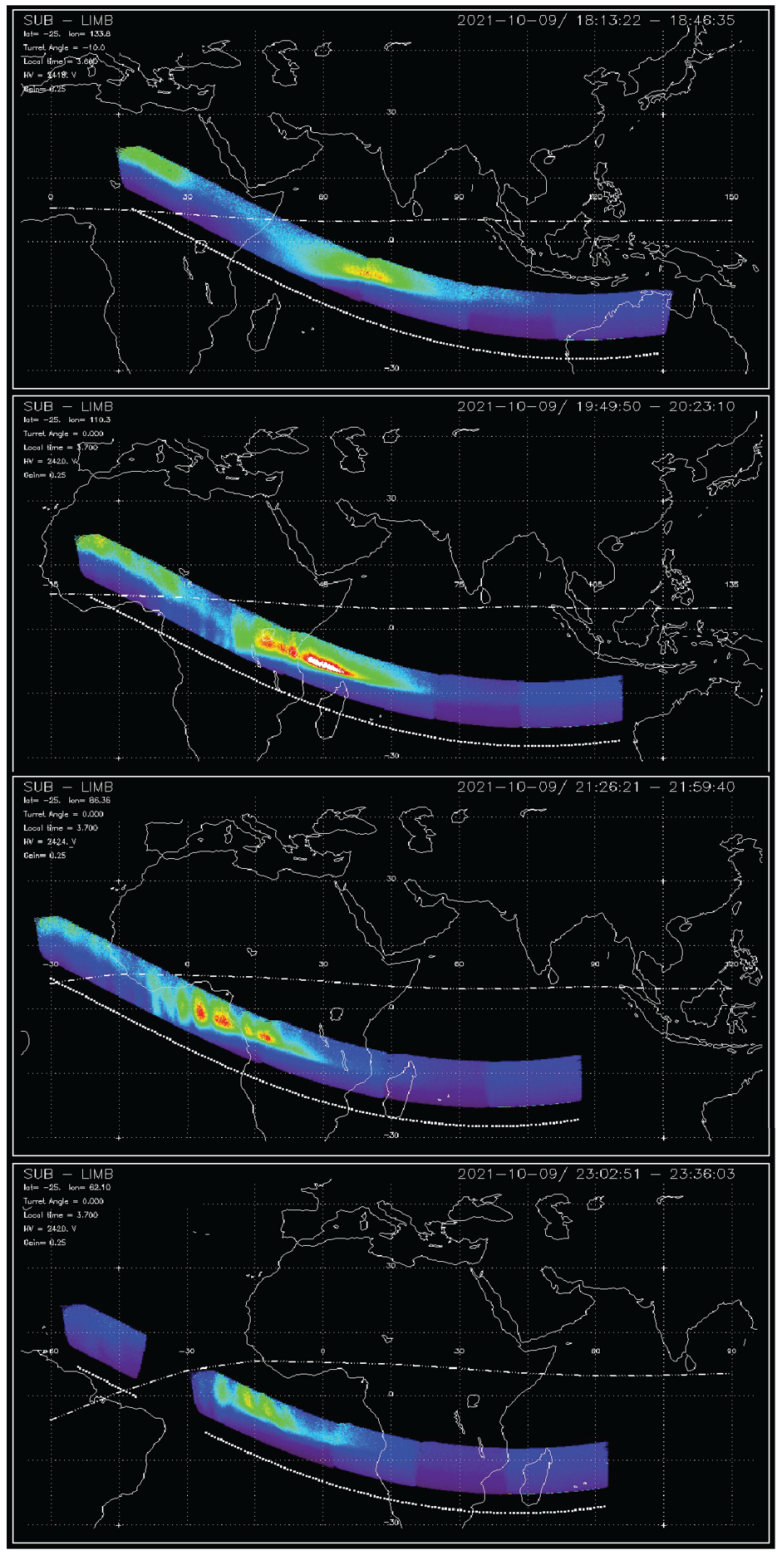
TDI imaging from the ICON FUV instrument at night, showing the distribution and changes in the equatorial ionosphere during four consecutive orbits of the observatory on October 9, 2021

A set of successive nighttime imaging frames can be combined, as shown for four successive orbits of October 2021 in Fig. 2. This figure shows the progression of the FUV orbit toward the west and the significant changes in the nighttime ionosphere that occur over that time. This imaging capability provides spatial resolution of better than 200 km across the nightside. The retrieval of nighttime ionospheric products has been validated against occultation retrievals from the COSMIC-2 mission (Schreiner et al. 2020) and networks of ground-based ionosondes (Wautelet et al. 2021), each of which provide similar products obtained solely through radio techniques, providing a completely independent reference for ICON FUV measurements.

**Fig. 3:**
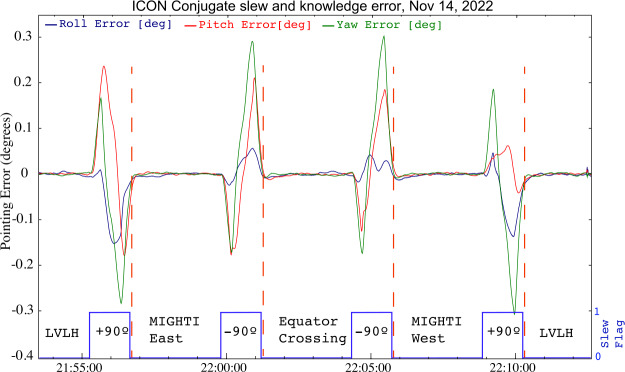
Pointing error of the ICON observatory during a full conjugate maneuver. As each of the four slews end, the pointing errors are converging and good MIGHTI and/or IVM data can be collected within 20 s of the completion of a yaw maneuver. (IVM is not in ram for MIGHTI East, West positions (Immel et al. 2018)

As noted earlier, ICON implements a two-part observational/operational strategy. The first part is a continuous sampling of conditions at the spacecraft by IVM and to the left of the orbit track by the three remote sensing instruments. IVM products are therefore always retrieved between ±27^∘^ geographic latitude, while the remote sensing products are offset northward by ∼15 degrees (in normal LVLH flight configuration). This constitutes more than 98% of the dataset collected by ICON. A second strategy is the conjugate maneuver, which in specific crossings of the magnetic dip equator rotates the spacecraft in three successive yaw rotations to collect observations to determine LoS winds to the north and south of the equatorial crossing position, and then returns to LVLH. It requires four ninety-degree yaw rotations and slew-and-settle to occur within 10 minutes, where the pointing performance settles down to required levels within 20 seconds of the completion of each rotation. An example of actual pointing system uncertainty during a conjugate maneuver is shown in Fig. 3. It is with these observations that ICON can provide the most complete comparison of wind drivers to vertical plasma drifts at the equator.

**Fig. 4:**
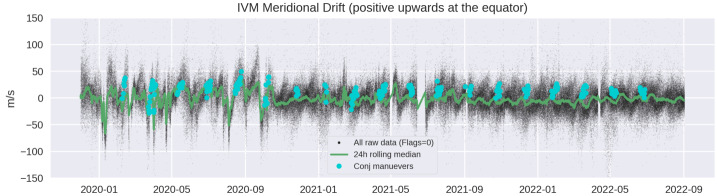
Time series of mean IVM daily meridional drifts (vertical at the dip equator) with instances of conjugate maneuvers shown with blue indicators

With selection of crossings at locations of large positive(negative) declination for descending(ascending) nodes of the orbit, the LoS winds are combined into cardinal wind products at locations falling near both northern and southern magnetic footpoints. Because locales with relatively large absolute declination provide the most relevant wind-plasma comparisons, these conjugate maneuvers are implemented over the Eastern Pacific and the Atlantic Oceans. The region of the South Atlantic Anomaly is avoided. These maneuvers have been implemented during every period that supported these observations since the start of the mission. When their occurrence is viewed over the entire mission (Fig. 4), one sees the intermittent nature of the maneuver because it is only performed in daytime and away from the terminators.

**Fig. 5:**
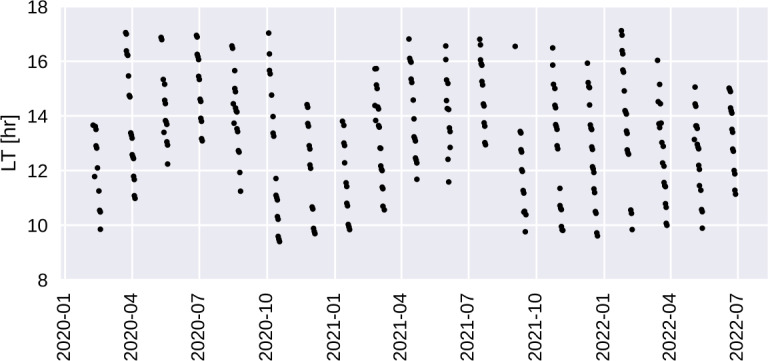
The occurrence of conjugate maneuvers during the mission is shown in mission elapsed time and local time

The local time of these operations over a two year period is shown in Fig. 5. Relevant to the distribution of these data is an operational flight rule to avoid science maneuvers in daytime during periods of high absolute orbital beta angle ($\beta > 35^{\circ}$ or $\beta < -35^{\circ}$) that was respected during the prime mission. High beta periods are marked by minima in net solar power generation per orbit, and so the rule prohibiting maneuvers assures the greatest possible solar generation during these periods. As a result the conjugate maneuver is performed outside of specific local times the range of which varies throughout the year. This can be seen as gaps at early and late local times in Fig. 5. After 2 years of operations, a review of the operation showed that the energy loss due to conjugate maneuvers is nearly insignificant, and the rule was waived for conjugate operations. At the end of the time in the figure, post-prime mission, one can see that the most recent conjugate maneuvers are performed over a full range of SLTs.

## Scientific Findings

### Objective 1: Day-to-Day Ionospheric Variability

**Fig. 6:**
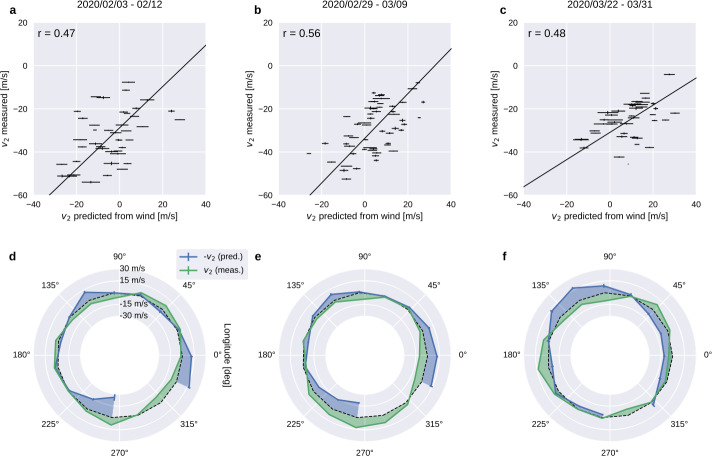
These are average IVM drifts and MIGHTI-based predictions of same over 3 successive precession periods where ICON’s orbit crossed noon at the magnetic equator. The zonal variation in both winds and drifts provides a range of physical conditions that support a correlation analysis that is shown with the best linear fit above each longitudinally organized comparison of the same data. The method by which the vertical drift ($v_{2}$) is predicted from the remote wind observations is discussed in detail in the original report (Immel et al. 2021)

The ICON winds and in-situ plasma drifts are combined in the first spaceflight investigation of the efficiency of neutral winds in driving electric currents that put the equatorial plasma into motion, causing it to grow into the densest reservoir of plasma in geospace. First-principles simulations predict related, large changes in the ionosphere, primarily through modification of wind-driven electromotive forces – the wind-driven dynamo. ICON provides the first direct evidence of the action of a wind dynamo in space, using the coordinated, space-based observations of winds and plasma motion, finding a clear relationship between the vertical plasma velocities measured at the magnetic equator near 600 km and the thermospheric winds much farther below. Significant correlations are found, during several successive precession cycles of ICON’s orbit, between plasma velocities observed near the equator and the same velocity calculated from wind measurements weighted by Hall and Pedersen conductivities (Immel et al. 2021), as shown in Fig. 6.

The outcome of ICON’s discovery is that prediction of thermospheric winds in the 100 – 150 km altitude range emerges as the key to improved prediction of Earth’s plasma environment. This is reinforced by the dual north-south measurements that come from the ICON conjugate maneuver, which has provided 359 complete, combined characterizations of the dynamo winds and drift. In each case where the southern footpoint winds are included in calculation of predicted drift, the correlation between the measured and predicted drift improves. This result is found by Harding et al. (2023), and further supports the finding that the key to prediction of the behavior of the equatorial ionosphere is in predicting what the E-region winds will be. The times where the 2.1 and 2.2 MIGHTI wind products together with the 2.7 IVM ion velocity product provide complete conjugate measurements are indicated in Fig. 4. All of these data products include attributes that identify the times of conjugate operations.

The day-to-day variability in the ionosphere often expresses itself in a strong modification of the strength and direction of the equatorial electrojet (EEJ) and associated vertical drift of plasma. Using MIGHTI data, Yamazaki et al. (2021) made the first direct comparisons of E-region winds to electrojet current measurements, finding that the magnitude and direction of winds in the 100-180 km range showed a strong statistical relation to the magnitude and direction of EEJ currents at lower altitudes. This was demonstrated again in grand fashion by the transit of a large thermospheric wave associated with the volcanic eruption in January 2022 in Tonga through the American sector where both ESA Swarm and ICON were operating, where the wave produced both extremely large eastward and westward currents in response to E-region winds that were among the largest observed during the mission (${>} 3\sigma $ excursions from reference winds) (Harding et al. 2022). Several follow up studies (noted in Sect. 3.4) are in work to understand the global reach of this large volcanic eruption on Earth’s space environment.

The influences of the E-region on the much more abundant plasma at the F-peak clearly depend on a range of inputs from 100-150 km altitude. A remarkable finding by ICON is that the previously identified strong and highly variable wind shears observed in this altitude range by means of chemiluminescent trail injections at night (Larsen 2002) are similarly prevalent in the daytime E-region (England et al. 2022; Yiğit et al. 2022). The different effects of strong winds at the altitudes of peak Hall conductivity vs. higher altitudes of greater Pedersen conductivity lead to an overall variability in the driving electric fields. Thus, the accurate prediction of F-region development will require simulations of high fidelity and altitude resolution to correctly resolve wind shears. The WACCM model has the resolution to, in principle, simulate such shears, but model-data comparisons are just now underway.

### Objective 2 - Seasonal Variations in Wave Forcing

The ICON thermospheric products are evaluated in coordination with plasma measurements to address the second objective of the ICON mission – to evaluate the relative contributions of dynamics, chemistry and dynamo driving in creating the ionospheric response to global-scale waves. Much of this work is the automated detection of atmospheric tides and generation of a global-scale lower boundary tidal forcing for the TIEGCM model run. ICON’s relatively low inclination provides the most rapid orbital precession to fully sample geographic and solar local time locations, while sampling a range of latitudes that is large enough to fully inform fits to the data that require this information.

**Fig. 7:**
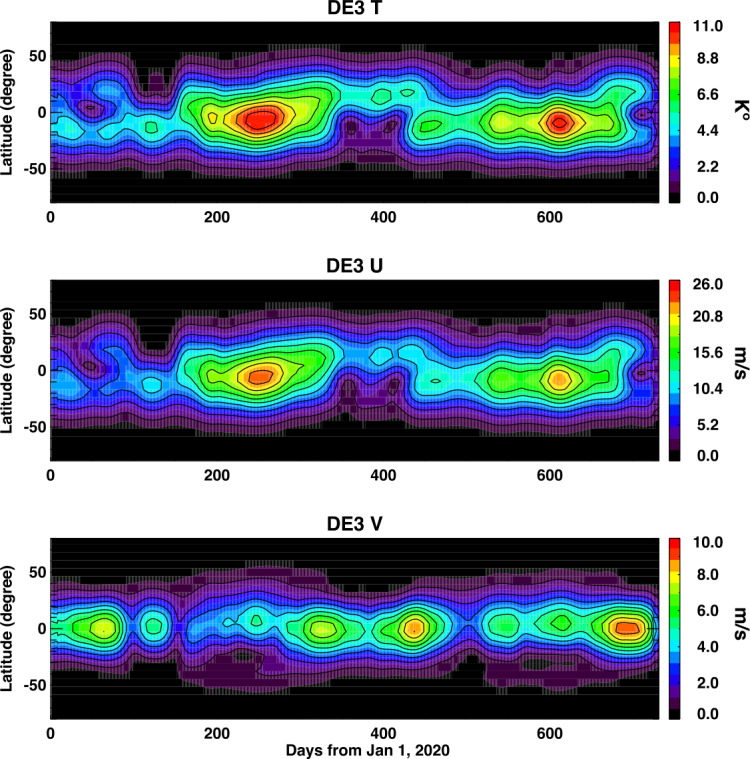
This is the amplitude of the Diurnal Eastward -3 tide (DE3) over the ICON prime mission seen in temperature, zonal, and meridional wind determined from the fit of the Hough-Mode Extensions to the wind and temperature data from ICON MIGHTI

One of the largest atmospheric waves that is observed in space is a tide energized by tropospheric and stratospheric heat sources: the diurnal eastward tide with a zonal wavenumber of 3 (DE3). Retrieval of the amplitude, phase and global distribution of tides like this is a key capability of ICON. The retrieval of this among a range of other diurnal and semi-diurnal tides is done through the use of an algorithm that fits a set of atmospheric Hough Modes to the temperature and wind data from MIGHTI (Forbes et al. 2017; Cullens et al. 2020). The result of that retrieval for the 2-year prime mission is shown in Fig. 7. Because this tide is energized in the lower and middle atmosphere, the interannual variation in the DE3 observed in space can clearly be attributed to year-to-year changes in weather and climate on Earth.

The full complement of remote sensing observations from ICON provided several other first-of-their-kind observations, including the first observational evidence for the effects of tides on thermospheric composition (England et al. 2021), a key to quantifying the dynamical influence of tides on ion chemistry. Other studies examined different potential coupling mechanisms. One study examined ionospheric variability produced during periods when both strong non-migrating tides and multi-day planetary waves are present (Forbes et al. 2021). Their results demonstrated that dynamical coupling of the thermosphere and ionosphere produce a complex pattern of response in the ion densities at F-region altitudes. In another analysis, (Forbes et al. 2021) daily variability in the DE3 and SPW4 atmospheric tides were identified using a quadrant-splitting approach to fully sample tides with effective zonal wavenumber of 4 on 24-hour timescales. This is verified by demonstrating in the same study that the IVM data show a similar daily density variability, providing an independent source that is known to be physically related for the fact of all the efforts on Objective 1 (Sect. 3.1). Two other reports (Liu et al. 2021; Gasperini et al. 2021) both identified strong dynamo coupling of atmospheric waves in the E-region that resulted in F-region ion density variations, with the latter focusing on a time period in which both ICON IVM and complementary cubesat data were available, permitting a cross-platform comparison of the tidal signatures. In a study of planetary wave signatures (He et al. 2021), it was determined for the first time that the quasi 2-day wave is in fact two planetary waves with periods smaller and larger than 48 h with different zonal wavenumbers. This is verified with new, simultaneous observations from a ground-based chain of radars in Asia that share in this discovery. Because of the continuous global sampling of winds and temperatures, the ICON data can characterize the tide with much higher spatial and temporal resolution than ground-based observatories.

**Fig. 8:**
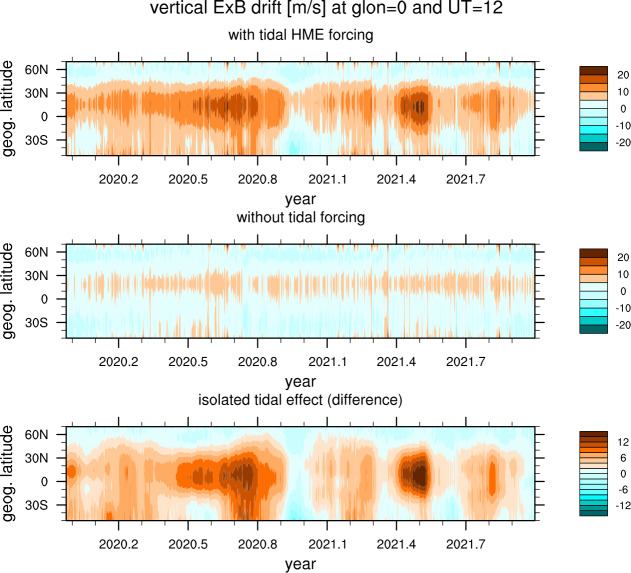
These are TIEGCM the vertical ExB drift in the $\mathrm{Lon} = 0^{\circ}$ meridian from the TIEGCM over the ICON prime mission. The top plot shows the plasma drift when incorporating lower atmospheric forcing. The middle plot is a baseline run without that forcing. The difference (bottom panel) isolates the remarkable effect of the lower atmosphere on the ionosphere

The diurnal and semidiurnal tides determined by ICON are all inputs to a 2-year run of the ICON TIEGCM (Maute 2017). They are included by means of the specification of the lower boundary of the model that is informed by the full HME set derived from ICON data. The vertical plasma drift at noon in the 0 deg W meridian is calculated by the TIEGCM and shown in the top panel of Fig. 8. For comparison, the TIEGCM is run with a simplified lower boundary which does not assimilate any ICON observations, and shown in the second panel of Fig. 8. The difference between these runs (bottom panel) shows the remarkable effect that the lower atmosphere has on vertical drifts of the ionospheric plasma.

**Fig. 9:**
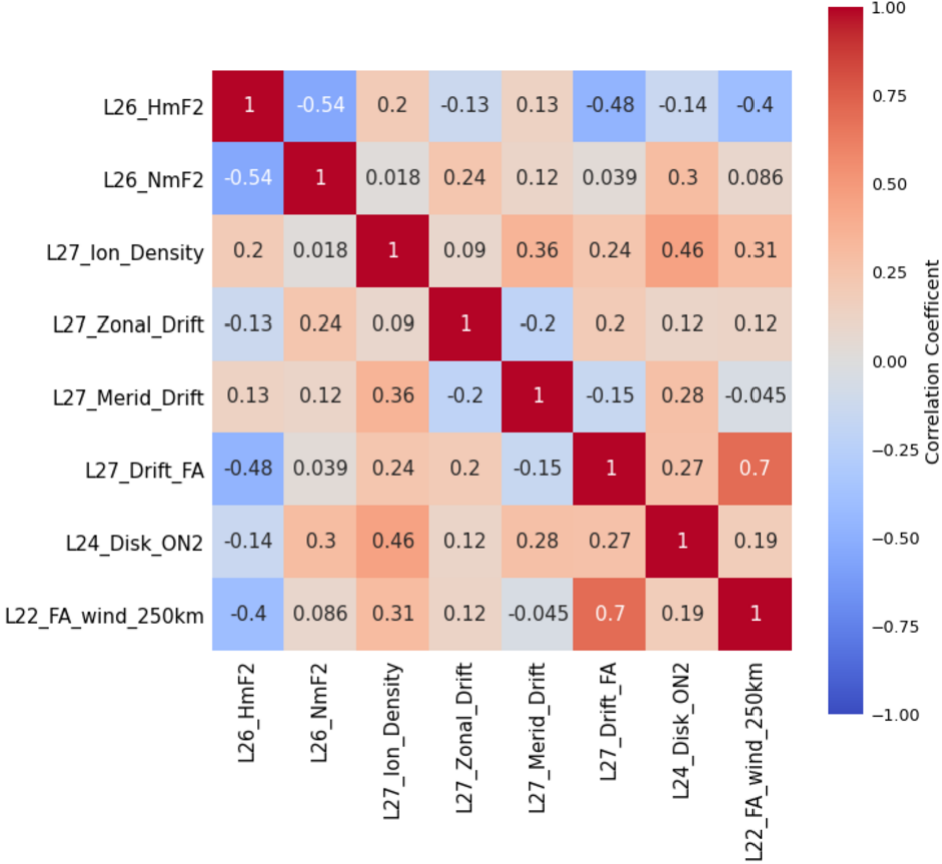
Correlation matrix of key ICON data products related to ionospheric density, velocity, and field-aligned neutral winds. The short hand abbreviations indicate products from MIGHTI (L22), FUV (L24), IVM (L27) and EUV (L26). All remote sensing observations (L22, L24 and L26) are retrieved parameters to the north of the observatory

Other approaches to analysis can be performed that do not concentrate on the retrieval of tides or other waves. The number of data products provided by ICON lend themselves to a broad search for relations between parameters. Using the most recent versions of products, we examine a set of available observations for 2020 made near the magnetic equator near noon. In Fig. 9, the cross-correlation of several key ICON products over an entire year is shown. This captures the relationships between key properties of the IT system as they are significantly influenced by the significant changes in season and tides through the year.

The highest correlation is between field-aligned drift and field-aligned neutral winds. The interhemispheric transport of plasma as coupled to the neutral winds is observed by ICON for the first time, and the correlative evidence is discussed in much greater detail in a recent study of the connection between the neutral atmosphere and plasma flow as they vary over the two years of the prime mission (Heelis et al. 2022). Connections such as these are possibly easier to evaluate during the solar minimum conditions encountered by ICON, when magnetospheric drivers of ionospheric plasma flow are minimized.

Other significant correlation pairs, such as the notable anti-correlation of the ionospheric heights and peak densities (HmF2 and NmF2) values retrieved from the EUV observations, are often at first counter-intuitive, but may indicate actual physical effects occurring as the plasma densities grow in the daytime. This quick review of ionospheric products suggests several avenues of research that new studies might undertake in the future.

With ICON’s focused capability of retrieving tides that originate in the lower and middle atmosphere, it should not be thought that tides that are generated in the thermosphere cannot be retrieved and evaluated as well. Such is the case with several particular semi-diurnal tides, that are shown in new research efforts using ICON data to originate in the thermosphere itself. A detailed study using ICON thermospheric wind measurements finds a significant population of semi-diurnal tides that are created in the upper atmosphere (Forbes et al. 2022).

### Objective 3 - Ionospheric Storms

The third objective of the ICON mission is to understand how the competing effects of enhanced F-region thermospheric winds, disturbance dynamo effects, and penetrating magnetospheric electric potentials drive changes in the ionosphere during geomagnetic disturbances. ICON was launched during the 2 months of lowest solar sunspot activity and solar radio flux of the solar minimum period. Because of the low occurrence rate of enhanced geomagnetic activity, and the large effects of day-to-day changes in the thermospheric winds, it is hard to identify enough storm periods for a comprehensive study that establishes stormtime behavior of the ionosphere with low statistical uncertainty. However, several studies are now underway that target specific isolated events that occurred during the prime mission, or draw out storm effects using statistical approaches to identify key drivers and processes.

**Fig. 10:**
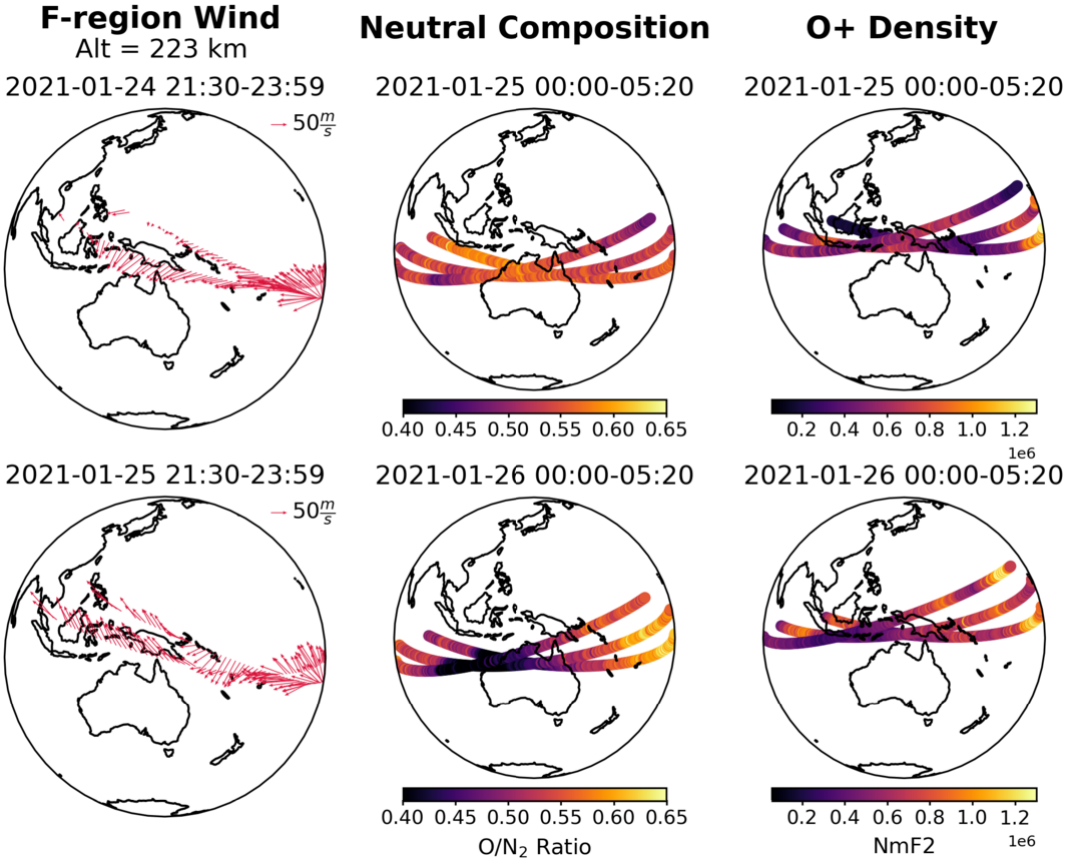
ICON products collected during the minor geomagnetic storm of January 24-26, 2021. Shown are 3 hours of wind products on successive days, prior to and after storm onset, are collected in early-to-late AM hours along the orbit track. The UV products come exclusively from daytime observations. A clear reduction of O/N_2_ is observed as well as O^+^ in the early morning, with a mix of enhancements/reductions in each of these parameters in the afternoon

In an ongoing case study (McGinness et al., in review), a small geomagnetic disturbance ($\text{minimum Dst} = -40~\text{nT}$) was found to be associated with significant, large changes in thermospheric composition and ionospheric densities. What is clear is that the ionospheric densities are driven to high levels by processes that are in competition with the chemical driver of strongly reduced O/N_2_ that is seen over a large area near the equator. In this event, equatorial plasma densities are affected by a period of enhanced high latitude magnetospheric inputs that produces strong equatorward winds and meridional transport of neutral species. This is deduced from the observation of several orbits of equatorward equatorial winds observed at low and middle latitudes. These results are summarized in Fig. 10. The strong northward change in meridional winds over the southern hemisphere carries a large composition bulge out of the auroral zone, but simultaneously contribute to enhanced densities by the increase in field-aligned winds (a notably effective driver of ion density as shown in Fig. 9).

**Fig. 11:**
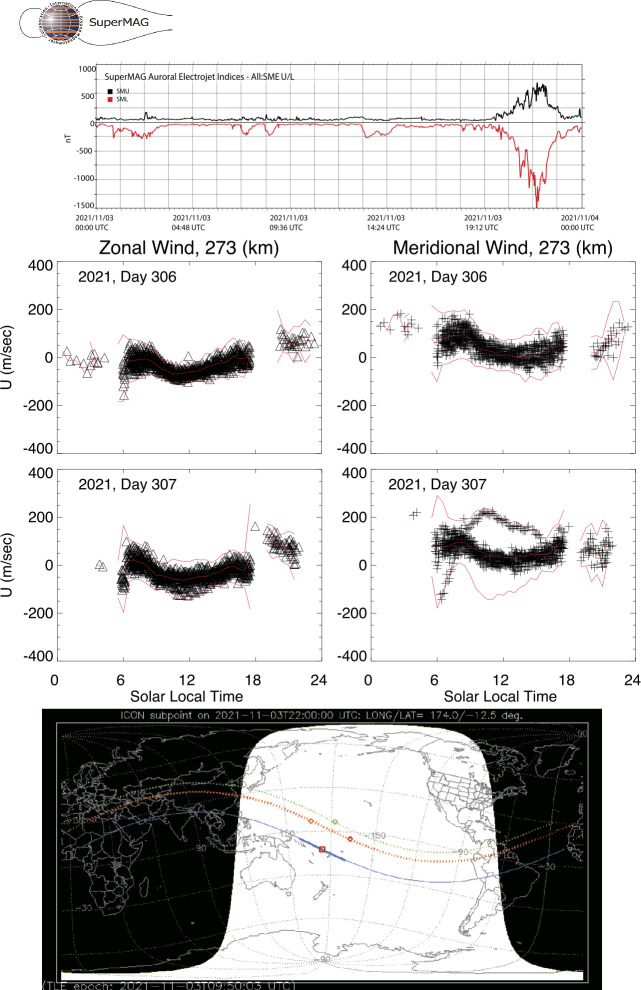
Thermospheric F-region winds during November 2, 3 (day 306, 307), 2021 that show a response to the onset of strong geomagnetic activity. Winds are shown near the top of the MIGHTI range @ 273 km altitude. Day 306 data are shown to establish reference mean winds and wind variance per hour. Following a rapid increase in auroral electrojet currents late on day 307, 2021 MIGHTI sees a significant (3$\sigma $) departure of the meridional wind from previous orbits, first with a southerly surge in the morning sector at northern middle latitudes, and a northerly surge in the late morning and afternoon at low latitudes. The blue trace in the map shows ICON’s locations during the orbit, and the red trace indicates the points of MIGHTI’s measurements

The redistribution of energy input at high latitudes leads to dramatic changes in the thermospheric winds, even at low and middle latitudes observed by ICON. Figure 11 shows an example of the abrupt changes in the winds seen in response to a minor geomagnetic storm that began late on November 3, 2021 early in the new solar cycle. A sudden onset of high latitude auroral currents and heating may be inferred from magnetic indices produced by the SuperMag project (Gjerloev 2012). The indices shown at the top of Fig. 11 indicate the envelope of eastward (SMU) and the stronger westward (SML) auroral electojet currents at high latitudes. These parameters are similarly constructed from ground magnetometer measurements as the World Data Center AU and AL indices (Newell and Gjerloev 2011) (except the SuperMag data are preliminary).

Average red line winds at 273 km altitude measured by ICON MIGHTI are shown for November 2 and 3, 2021 in the second and third rows of Fig. 11, where multiple passes are overlain to show the range of values observed both in zonal and meridional winds, with 3$\sigma $ range of samples in hourly bins shown in red. There is one orbit of data at the end of the day on Nov 3 that is a remarkable outlier, showing strong southward winds in the morning sector that transition to strong northward winds at noon and afternoon. These are clearly attributable to large-scale gravity waves originating in enhanced auroral inputs, with wind outliers in the meridional wind greater than 100 m/s. The zonal winds are much less affected, owing to the meridional propagation of waves, though a westward deviation evident near noon is consistent with southerly transport of gas by the waves, which through conservation of momentum (Coriolis force) will develop a westward velocity component. Whether this is a transient effect or indicative of a change in the large scale circulation of thermospheric neutrals is one of the topics of this study (McGinness et al. 2023). ICON’s observations include multiple examples of these events, with effects apparent with nearly every significant auroral heating event. With further research, critical new understanding can be developed and provide a pathfinder for some of the science targets for the upcoming NASA Geospace Dynamics Constellation (GDC) mission.

### New Science Investigations

The data that ICON provides has been used extensively in studies of the space environment in areas of interest outside of the original focused objectives of the mission. One area of investigation is the variability in the composition of the topside ionosphere. During the prime mission, ICON’s orbit is continuously above the F-layer peak, and at these altitudes the IVM instrument measures a significant proportion of H^+^. ICON provides the data to map the relative abundance of O^+^ and H^+^ ions, where variations in their density and relative abundance provide keys for understanding creation and loss of the plasma in the exosphere. This specific topic has particular importance at different local times, with studies focused on early morning (Huba et al. 2021), and post-noon (Park et al. 2021) changes in the two populations to advance understanding of the behavior of ionospheric plasma.

ICON enabled additional investigations of the topside ionosphere. The first paper focused on the variations in ion and electron temperatures measured by IVM near sunrise in coordination with the Jicamarca incoherent scatter radar (Derghazarian et al. 2021), where a second paper investigated the FUV emission-producing effects of early morning photoelectron fluxes flowing downward along magnetic field lines from magnetically-conjugate sunlit regions (Urco et al. 2021). This study suggested a list of corrections to the photoelectron component of the SAMI2-PE ionospheric model (Varney et al. 2012), while a latter study (Urco et al. 2021) identified an up to 20-30% 135.6-nm radiance contribution to nighttime recombination emissions, enabling the identification and removal of these emissions, which are surprisingly abundant in nighttime observations, prior to retrieval of O^+^ density profiles. This latter FUV study included GOLD observations as well, finding an initial offset bias multiplier of 0.5 between simultaneous GOLD and ICON measurements, with the ICON intensities being lower than GOLD. This discrepancy is addressed with the update of the ICON FUV Level 1 product to version 5.0.

The third topside paper focuses on low and middle latitude plasma irregularities, and investigate the correlation of their density structure with velocities and temperature, all measured by ICON IVM and the previously flown ROCSAT IVM (Park et al. 2021). The combination of extensive time spent by ICON in the equatorial ionosphere and the near-circular altitude provides an ideal dataset for addressing questions about the physics of ionospheric irregularities.

Impulsive forcing of the geospace environment by singular terrestrial events became a leading topic of research into ionospheric dynamics after the initial papers on tidal and planetary wave impacts on the global ionosphere (Immel et al. 2006; Goncharenko et al. 2010) brought new attention to the coupling of terrestrial process into space. The catastrophic 2011 Tohoku Earthquake produced a major signal in ionospheric densities that propagated around the world (Saito et al. 2011), and it was demonstrated that ionospheric measurements could provide key characteristics of the surface displacement in regions not instrumented with ground motion sensors (Astafyeva et al. 2011). The large body of work served as a progenitor of the studies now underway regarding the impacts of the 2022 Tonga volcanic eruption, now accompanied by neutral wind measurements provided by ICON (Harding et al. 2022; Aa et al. 2022; Le et al. 2022; Gasque et al. 2022; Vadas et al. 2023). ICON is playing a key role in characterization of the atmospheric waves generated by the eruption, while simultaneously providing direct observations of the global ionospheric effects of those waves.

### Summary

In its 3-year mission the ICON observatory performed very well and provided the data required to address the scientific goals and objectives defined in the early phases of mission development. ICON has enabled a series of remarkable findings described in this paper that have significantly advanced the fields of Earth and Space Science. The data products clearly have applicability to resolving other outstanding science questions beyond the original scope of the mission, as demonstrated by a number of new research projects already underway. The data are publicly available upon verification in the Science Data center where data continue to be processed to the time of the end of the mission in November 2022. Refinements in retrieval algorithms and data products continue to be implemented. The ICON Science Team remains prepared to support any new investigators and investigations into the properties of the IT system as it is affected by terrestrial and solar drivers.

### Epilogue

In the course of the development of ICON, there were a number of major contributors to the mission outside of the science team. To name a few, the success of the mission reaching orbit with full scientific and technical capability, and with the ability to perform in rLVLH, is due in part to the unyielding effort and skill of Drs. William Craig and Ellen Taylor, Project Manager and Systems Engineer, respectively, and Mr. Stuart Harris, lead electrical engineer, all at UC Berkeley. The excellent work by our instrument partners at Naval Research Laboratory and at Univ. Texas at Dallas, our payload integration partners (and more) at Space Dynamics Laboratory, and the support of the Explorers Office at Goddard Spaceflight Center all led to outstanding outcomes for the mission. Our partners at the Centre Spatial de Liège provided excellent support for the environmental test and calibration of the ICON FUV instrument. Finally our spacecraft provider at Orbital, Orbital ATK, and Northrop Grumman, led initially by Ms. Ann Cox, provided a high performance bus and were excellent partners.

The NASA Standing Review board provided excellent oversight, led by Mr. Rick Fitzgerald (APL). We benefited greatly from contributions from this board that included Mr. William Gibson (SwRI) and Mr. Steven Scott (GSFC), who both very sadly passed away before the launch of the mission. In our time spent working with them, we learned they found the ICON mission to be technically challenging but also of compelling importance to the scientific community. The mechanical engineering lead for the ICON instrument control package was Mr. Bill Donakowski, who saw delivery of the ICP but tragically passed away before launch. To these great engineers that we have lost, we are forever indebted.
